# Dual transcriptome of *Streptococcus mutans* and *Candida albicans* interplay in biofilms

**DOI:** 10.1080/20002297.2022.2144047

**Published:** 2022-11-09

**Authors:** Yan Zeng, Elena Rustchenko, Xinyan Huang, Tong Tong Wu, Megan L. Falsetta, Jin Xiao

**Affiliations:** a Eastman Institute for Oral Health, University of Rochester Medical Center, Rochester, NY, USA; b Department of Biochemistry and Biophysics, University of Rochester Medical Center, Rochester, NY, USA; c Department of Biostatistics and Computational Biology, University of Rochester Medical Center, Rochester, NY, USA; d Departments of Obstetrics and Gynecology and Pharmacology and Physiology, University of Rochester Medical Center, Rochester, NY, USA

**Keywords:** Biofilms, dental caries, transcriptome, *streptococcus mutans*, *candida albicans*, oral infection

## Abstract

**Objective:**

To assess the interactions between *Streptococcus mutans* and *Candida albicans* during cariogenic biofilm formation.

**Methods:**

The *S. mutans* and *C. albicans* duo-species biofilms were formed in 1% sucrose to mimic the high caries risk challenges. The biofilm structure was assessed using two-photon laser confocal microscopy. The transcriptome of 48h-biofilms was assessed by RNA-Seq. The expression of *S. mutans* and *C. albicans* virulence genes was examined via real-time reverse transcription-polymerase chain reaction.

**Results:**

The morphogenesis of *C. albicans-S. mutans* duo-species biofilms was significantly altered when comparing to *S. mutans* or *C. albicans* single-species biofilm. Duo-species biofilms exhibited unique expression profile with a large number of differentially expressed genes (DEGs), including a higher expression of *S. mutans atpD* (acid-adaptive), *C. albicans CHT2* (fungal cell wall chitin remodeling), and *C. albicans SOD3* (cytotoxic oxygen radical destroying) (p < 0.05). KEGG pathway analyses further revealed that the majority of the up-regulated DEGs are related to microbial metabolism. Furthermore, the expressions of *S. mutans* and *C. albicans* key virulence genes (*gtfB*, *gtf*C, *gtfD*, *ECE1*, *HWP1*, *ERG4*, *CHT2*) were associated with sugar availability-related and time-related dynamics.

**Conclusion:**

Cross-kingdom interactions impact *S. mutans-C. albicans* biofilm formations and dynamic expressions of virulence genes.

## Introduction

Microbial interactions are crucial to maintaining microbial populations, microbiome structure, and ecosystem functions [1–3]. Ranging from mutualism to antagonism, interactions between bacteria and fungi have been in the spotlight because they play an essential role in driving biochemical cycles, maintaining balance in numerous ecosystems, and contributing to health and disease [4,5]. Interaction mechanisms have been elucidated for several pathogenic bacteria–fungi relationships, such as *Candida albicans* and the commonly isolated bacterial species *Pseudomonas aeruginosa* and *Staphylococcus aureus* [6], the microbial secondary metabolite-mediated interaction between the plant-pathogenic bacterium *Ralstonia solanacearum* and two plant-pathogenic fungal organisms *Fusarium fujikuroi* and *Botrytis cinerea* [7], and the characterization of 16 different bacterial-fungal pairs, examining the impact of 8 different fungi isolated from cheese rind microbiomes and two bacterial species (*Escherichia coli* and a cheese-isolated *Pseudomonas psychrophila*) [1].

Bacteria *Streptococcus mutans* and fungi *Candida albicans* are considered pathogenic microorganisms responsible for causing and accentuating oral diseases [8]. For instance, previous studies support the clinical importance of the association between *S. mutans* and *C. albicans* in the pathogenesis of early childhood caries (ECC) [9,10]. This relationship fosters initiation and maturation of cariogenic biofilms/plaque on the tooth surface, mediating cariogenic development [11,12], and ultimately causing ECC. Furthermore, our previous study examined the oral colonization of *S. mutans* and *C. albicans* among a cohort of underserved infants and indicated the early colonization of oral *C. albicans* is associated with a 3.5 times higher risk of *S. mutans* emergence when children turn one year of age. We also revealed a positive correlation between infant salivary *S. mutans* abundance and the *C. albicans* colonization [10].

In addition to clinical evidence of the symbiotic relationship between *S. mutans* and *C. albicans, in vitro* biofilm studies and animal models further support their collective role in caries disease [12–16]. A rat model showed that *S. mutans* and *C. albicans* co-infection led to a rapid onset of severe carious lesions on the smooth surfaces of teeth. The presence of *C. albicans* and *S. mutans* dramatically enhanced the assembly of an exopolysaccharide (EPS)-rich matrix, leading to the development of thicker biofilms than those formed by *S. mutans* or *C. albicans* alone [12]. Moreover, the duo-species biofilm matrix of *S. mutans* and *Candida spp*. delayed or blocked the antimicrobial diffusion into the biofilm, making treatment much more difficult or unsuccessful [17], dual-species biofilms are more resistant to stress conditions [16].

While attention has been devoted to examining the synergistic relationship between *S. mutans* and *C. albicans*, the molecular basis of *S. mutans* and *C. albicans* interplay, especially global transcriptomic analysis, remain under-investigated. Although transcriptome analysis of *S. mutans* in duo-species biofilms (i) confirmed the induction of specific genes (such as *comS, sigX*) and (ii) the stimulation of the complete quorum sensing system of *S. mutans* by *C. albicans* [18], as well as (iii) demonstrated a dramatically altered gene expression in *S. mutans* in the presence of *C. albicans*, however, the dual transcriptome of *S. mutans* and *C. albicans* interplay in duo-species biofilms remain unclear [19]. To better understand the microbial interactions between *S. mutans* and *C. albicans* during cariogenic biofilm development, we used an *in vitro* biofilm model that mimics a clinical high caries risk condition. We performed transcriptomic analysis via RNA-Seq and real-time reverse transcription-polymerase chain reaction (qRT-PCR) on single- and duo-species biofilms. Our results revealed distinctive transcriptomic profiles of *S. mutans* and *C. albicans* in duo-species biofilm development compared the single-species biofilms. We also revealed the dynamic expression of several virulence genes in biofilms and culture media. This knowledge may be vital to developing better strategies to prevent and treat ECC.

## Materials and methods

### Bacterial strains and starter preparation

*S. mutans* UA159 and *C. albicans* SC5314 were recovered from frozen stock using Blood agar (TSA with Sheep Blood, Thermo Scientific™ R01202) and YPD agar (BD Difco™, 242,720), respectively. After a 48-h incubation, 3–5 colonies of each species were inoculated into 10 ml of broth for overnight incubation (5% CO2, 37°C). *S. mutans* was grown in TSBYE broth (3% Tryptic Soy, 0.5%Yeast Extract Broth, BD Bacto™ 286,220, and Gibco™ 212,750) with 1% glucose; *C. albicans* was grown in YPD broth (BD Difco™, 242,820). The following morning, 0.5 ml of the overnight starter was added to the fresh broth and incubated for 3–4 h until reaching the mid-exponential phase with desirable optical density (the optical density at a wavelength of 600 nm (OD600) of 1.0 for *S. mutans* and OD600 of 0.8 for *C. albicans*). The morning starters were used for biofilm formation.

### Biofilm models

**Figure 1.**
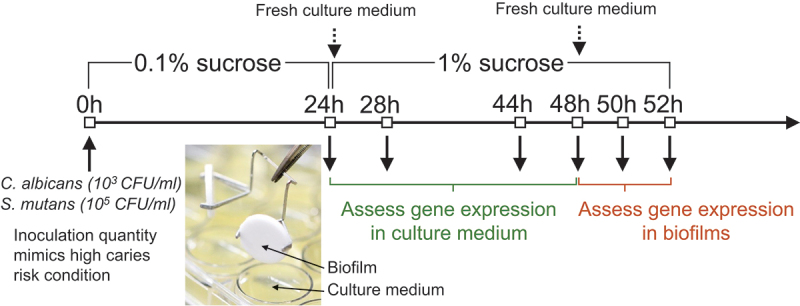
**Study design** The saliva-coated hydroxyapatite disc, consisting of similar components as in tooth enamel, was used as the substrate for biofilm formation. *C. albicans* and *S. mutans* were grown alone or together in 0.1% sucrose without disturbance until 24-h for initial biofilm establishment. The culture medium was changed once daily. To induce a cariogenic challenge, the culture medium was changed to 1% sucrose at 24-h and 48-h. The inoculation quantity of *C. albicans* (10^3^ CFU/ml) and *S. mutans* (10^5^ CFU/ml) was chosen to mimic high caries risk conditions in the clinical setting.

We used a duo-species biofilm model to assess the interactions between *S. mutans* and *C. albicans* during biofilm formation; see Figure 1 for study flow. Single species biofilms of *S. mutans* or *C. albicans* alone were grown as controls. The biofilm method detailed previously [20,21] formed on the saliva-coated hydroxyapatite discs (0.50” diameter × 0.05” thickness, Clarkson Chromatography Products, Inc., South Williamsport, PA) was employed. The discs were placed vertically in a 24-well plate using a custom-made disc holder to mimic the caries-prone smooth tooth surfaces in the oral cavity [20]. The inoculation quantity of *S. mutans* [10^5^ colony-forming unit (cfu)/ml] and *C. albicans* (10^3^ cfu/ml) mimicked a high-risk clinical condition. Biofilms were formed in 2.8 ml of TSBYE broth with 0.1% (w/v) sucrose in a 24-well plate. The organisms were grown undisturbed to allow initial biofilm formation during the first 24 h at 5% CO_2_ and 37°C. At 24-h and 48-h, biofilms were transferred to a fresh cultural medium with 1% (w/v) sucrose to induce cariogenic challenge. Biofilms were collected at selected time points for biological (biofilm structure and cultural medium pH) and molecular (RNA-seq and qRT-PCR) assays. Independent assays were conducted three times with four replicated discs in each round.

### Laser scanning confocal fluorescence microscopy (LCSFM) imaging of biofilm matrix

We assessed two essential components of the biofilm matrix: bacteria and exopolysaccharides (EPS) using LCSFM methods detailed previously [18]. Briefly, 1 μM Alexa Fluor® 647-labeled dextran conjugate (Molecular Probes, Invitrogen Corp., Carlsbad, CA) was added to the culture medium from the beginning of and during the development of the biofilm to enable visualization of exopolysaccharides. The bacterial and fungal species were labeled by SYTO® 9 green-fluorescent nucleic acid stain (485/498 nm; Molecular Probes). The images were obtained using an Olympus FV 1000 two-photon laser scanning microscope (Olympus, Tokyo, Japan) equipped with a 10X (0.45 numerical aperture) water immersion objective lens [22]. Amira 5.0.2 (Mercury Computer Systems Inc., Chelmsford, MS) was used to create 3D renderings of EPS and bacteria of the biofilms detailed previously [22,23].

### Transcriptome analysis by RNAseq

The 48 h biofilm formed by single-species (*S. mutans* or *C. albicans*) and duo-species (*S. mutans*+*C. albicans*) underwent RNA-seq analysis. The biofilms were harvested from four discs for each condition. The discs were incubated in RNALater (Applied Biosystems/Ambion, Austin, TX, USA) for 1 h before biomass collection. RNA extraction and purification were performed with MasterPure complete DNA and RNA purification kit (Epicenter, Lucigen, Wisconsin, USA). Raw RNA product was quantified by NanoDrop One Microvolume UV-Vis Spectrophotometer (Thermo Scientific™, Wilmington, DE, USA). The rRNAs were depleted with Ribozero rRNA Removal Kit (Illumina, San Diego, CA, USA). An RNA sequencing library was prepared using NEBNext Ultra RNA Library Prep Kit for Illumina by following the manufacturer’s recommended protocol (NEB, Ipswich, MA, USA). Briefly, enriched RNAs were fragmented at 94°C for 15 minutes. The cDNA fragments were end-repaired and adenylated at 3ʹends, and a universal adapter was ligated, followed by index addition and library enrichment with limited cycle PCR. Sequencing libraries were validated using the Agilent TapeStation 4200 (Agilent Technologies, Palo Alto, CA, USA) and quantified by using a Qubit 2.0 Fluorometer (Invitrogen, Carlsbad, CA) as well as by quantitative PCR (Applied Biosystems, Carlsbad, CA, USA).

Following the manufacturer’s instruction, the sequencing libraries were multiplexed and clustered on one flow cell lane and loaded on the Illumina HiSeq instrument. A 2 × 150 Paired-End (PE) configuration was used for sample sequencing. HiSeq Control Software (HCS) was used for image analysis and base calling. Raw sequence data generated from Illumina HiSeq was converted into FASTQ files and de-multiplexed using Illumina’s bcl2fastq 2.17 software. The sequence reads of all samples were deposited in the NCBI Sequence Read Archive (SRA) as a study under the accession number of PRJNA809829. One mismatch was allowed for index sequence identification. The sequence reads were trimmed using Trimmomatic v.0.36 and were mapped by the STAR aligner v.2.5.2b [24] to the reference genomes. Unique gene hit counts were calculated using feature Counts from the Subread package v.1.5.2. Unique reads within exon regions were counted. Gene hit counts were extracted, and the gene hit counts table was used for downstream differential expression analysis.

A comparison of gene expression between the groups of samples was performed using DESeq2. p-values and Log2 fold-changes were calculated by the Wald test. *S. mutans* genes with adjusted p-values (False Discovery Rate (FDR) p-values) <0.05 and absolute log2 fold changes >1 and *C. albicans* with adjusted p-values (False Discovery Rate (FDR) p-values) <0.05 and absolute log2 fold changes >3 were defined as differentially expressed genes (DEGs) for each comparison. The statistically significant genes underwent a gene ontology (GO) analysis by implementing the software GeneSCF v1.1 [25]. The GO list was used to cluster the set of genes based on their biological process and determine their statistical significance. The ‘plotPCA’ function within the DESeq2 R package was used for performing Principal component analysis (PCA); the first two principal components were plotted in a 2D plane for the samples. The top 500 genes, selected by highest row variance, were used to generate the plot. Volcano plots were generated by VolcaNoseR (https://huygens.science.uva.nl/VolcaNoseR) [26]. Kyoto Encyclopedia of Genes and Genomes pathways were generated by KEGG mapper (genome.jp) and Cytoscape software version 3.8.2.

### Real-time reverse transcription polymerase chain reaction assay

qRT-PCR experiments were performed to validate genes of interest and specific genes associated with the virulence factors or viability of *S. mutans* or *C. albicans*. The genes and primers used are listed in Table S1. Methods were detailed previously [20].

In each experimental condition, RNAs in biofilms were collected and extracted from four discs, and RNAs in the culture medium were collected and extracted from the liquid of two wells (4 ml) from the 24-well plate. 0.2 µg of purified RNA was used as a template for cDNA synthesis with the BioRad iScript cDNA synthesis kit (Bio-Rad Laboratories, Inc., Hercules, CA). cDNA and negative control samples were amplified using Applied Biosystems™ PowerTrack™ SYBR Green Master Mix and a QuantStudio™ 3 Real-Time PCR System (Thermo Fisher Scientific, USA). Each 20 µl reaction contained cDNA, 10 µM of each primer, and 2× SYBR-Green mix (SYBR-Green and Taq DNA Polymerase). *gyrA* for *S. mutans* genes [27] and *ACT1* for *C. albicans* were used as the internal reference for comparative expression calculation. Data analysis was conducted using the comparative CT method [28].

Furthermore, to determine the dynamic changes of genes of interest during duo-species biofilm formation, we performed qRT-PCR assays for biofilms and culture medium at specific stages. The time points selected for gene expression assessment were before and after culture media change associated with a change in sugar availability. For the schematic design, see Figure 1.

### Statistical analysis

Dynamic expression of genes was assessed by qRT-PCR. For the genes in biofilms, 2^ (-ddCT) value of each specific time point was compared with that at 48-h. For the genes in the culture medium, 2^ (-ddCT) value of each specific time point was compared with that at 24-h. Normality tests were first performed. When comparing the duo-species biofilm to the single-species biofilm, the comparison of the 2^ (-ddCT) values was assessed using the t-test for normally distributed data and the Mann-Whitney U test for non-normal data. When comparing the gene expression of different time points among the duo-species biofilms and culture medium, repeated ANOVA with a post hoc test was used after assessing data normality. Statistical tests were two-sided, with a significant level of 5%. IBM SPSS was used for statistical analyses.

## Results

### Presence of both *S.* *mutans* and *C.* *albicans* enhanced biofilm formation

**Figure 2.**
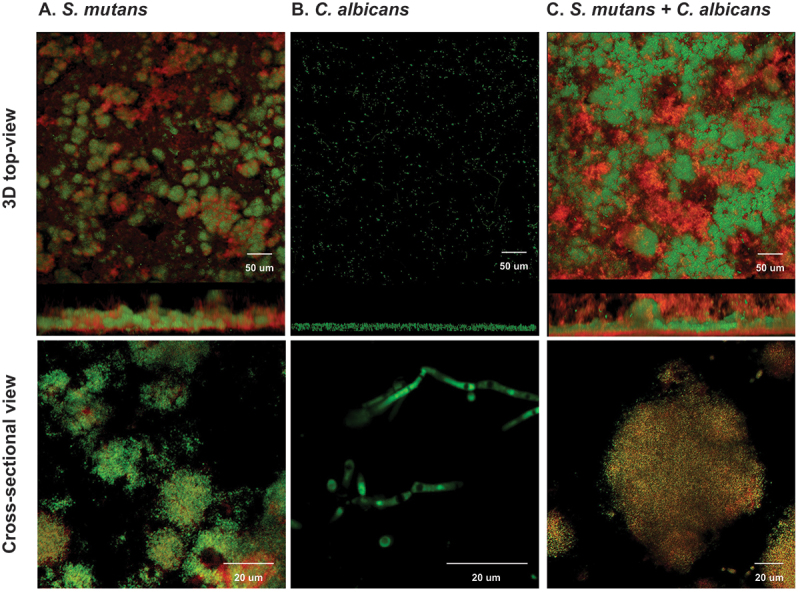
**Morphogenesis of micro-colonies in 48-h biofilms (1% sucrose condition)** In 1% sucrose condition, the 48-h biofilms were visualized by a two-photon laser confocal microscope. The biofilms of single species were shown in panel A (*S. mutans*) and panel B (*C. albicans*). The biofilms of duo-species (*C. albicans* and *S. mutans*) was shown in panel C. The confocal images indicate the cross-sectional and 3D-top views of biofilms; the green color indicates bacteria, and the red indicates the exopolysaccharides (EPS). Compared to the single species, the morphogenesis of *C. albicans* and *S. mutans* duo-species biofilms were significantly altered and characterized by formation of well-structured microorganism cluster enmeshed with EPS. These clusters are defined as microcolonies. In the biofilm formed by *C. albicans* alone, no microcolonies defined as above were identified.

The 48-h duo- and single-species biofilms were visualized by a two-photon laser confocal microscope. The 3D top and cross-sectional views of microcolonies are shown in Figure 2. Compared to the single species (Figure 2A and 2B), the morphogenesis of *C. albicans-S. mutans* duo-species biofilms (Figure 2C) was significantly altered. Microcolonies in biofilms are considered virulent and functional structures. Larger micro-colonies and significantly more EPS were formed in duo-species biofilms when compared to *S. mutans* single species biofilms. Well-formed mushroom-shaped microcolonies were identified grown in the duo-species group (Figure 2C). By comparison, microcolonies formed by *S. mutans* alone were less structured, with less bacteria content enmeshed with EPS (Figure 2A). Not surprisingly, few microcolonies and barely no EPS were formed in the *C. albicans* single-species biofilms; *C. albicans* does not form biofilms well alone under the selected culture conditions (Figure 2B).

### Transcriptome analysis reveals interactions between *S.* *mutans* and *C.* *albicans* in biofilms

The Principal Component Analysis (PCA) (Fig S1) and the hierarchical clustering analysis (HCA) (Fig S2) indicate distinctive transcriptomic profiles of *S. mutans* and *C. albicans* in duo-species biofilm compared to those in single-species biofilm. Our data on the Q-Score distribution of RNA samples (Fig S3) support the validity of downstream analyses. The results of PCA and HCA also indicate high levels of correlation and reproducibility within the same sample types (single- and duo-species biofilms).

**Figure 3.**
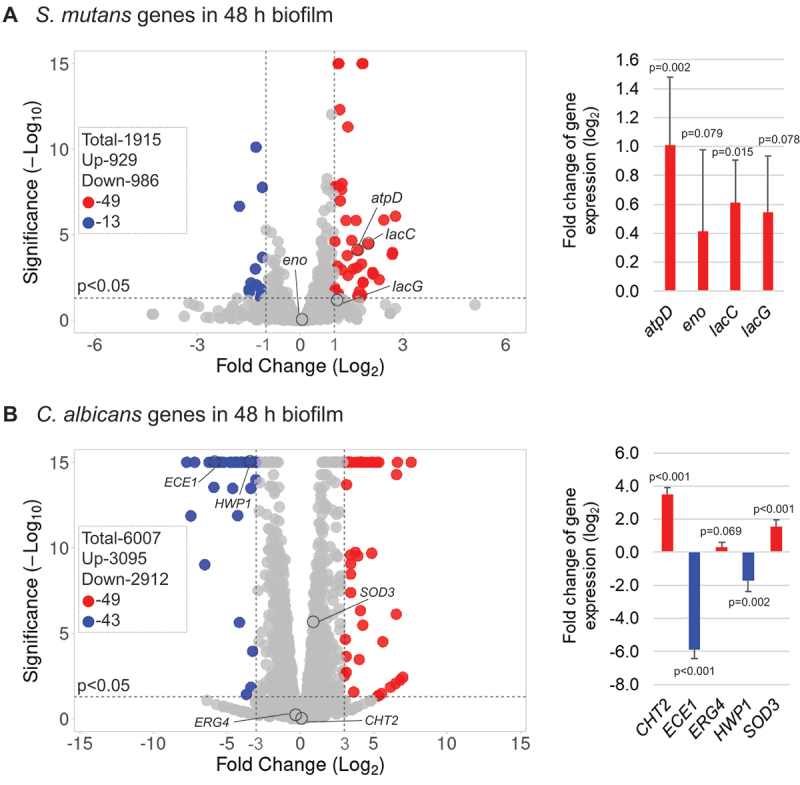
**Comparison of transcriptomic profiling between duo-species biofilms with single-species biofilms** (A) Volcano plots from transcriptome analysis of *S. mutans* in duo- (*S. mutans* + *C. albicans*) biofilm (48-h, 1% sucrose) compared to *S. mutans* in single-species biofilm. (B) *C. albicans* in duo-species biofilm (48-h, 1% sucrose) compared to *C. albicans* in single-species biofilm. Data represent three independent biological replicates of each condition. qRT-PCR validation results of selected genes are shown on the right side of each volcano plots.

Volcano plots in Figure 3 indicate the transcriptomic comparison between duo- and single-species biofilms. Overall, 1915*S. mutans* and 6007 *C. albicans* genes were detected via RNA-seq. Among the detected genes, nearly half of the *S. mutans* and *C. albicans* genes were up-regulated in duo-species biofilms. We further assessed the DEGs [> (-) 1 Log2 fold change] of *S. mutans* genes; 49*S. mutans* DEGs were up-regulated and 13 were down-regulated. We also assessed the DEGs [> (-) 3 Log2 fold change] of *C. albicans* genes; 49 *C. albicans* DEGs were up-regulated and 43 were down-regulated. The qRT-PCR validation for selected genes of interest was consistent with the RNA-Seq data. The *S. mutans* virulence genes in the context of cariogenicity were up-regulated when *S. mutans* grew with *C. albicans*. The genes include *atpD* (related to acid stress tolerance response), *eno* (related to the degradation of carbohydrates via glycolysis), and *lacC* and *lacG* (related to galactose metabolism). Similarly, when grown with *S. mutans, C. albicans* genes related to fungal cell wall chitin remodeling (*CHT2*) and cytotoxic oxygen radicals destroying (*SOD3*) were significantly up-regulated [3.5 Log2 fold increase for *CHT2* (p = 0.01), and 1.5 Log2 increase for *SOD3* (p = 0.02)]. However, our results show that *C. albicans* hyphal-specific *ECE1* were down-regulated in duo-species biofilms.

**Figure 4.**
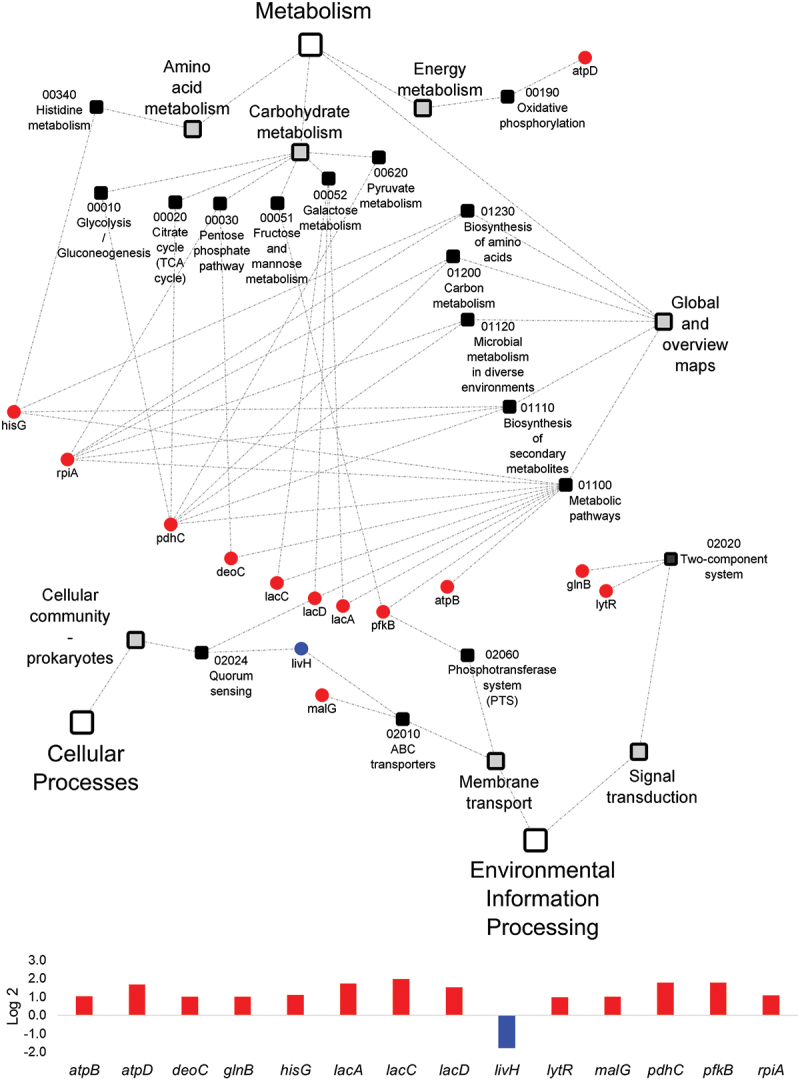
**KEGG pathway network for *S. mutans* differentially expressed genes between the duo-species and single-species biofilms** The genes of *S. mutans* that are differentially expressed between the comparison groups with FDR p-values < 0.05 and log2 fold changes > 1 were defined as *S. mutans* DEGs and listed in Supplementary Table 4. 17 impacted pathways were found for *S. mutans* DEGs. The fold change of the DEGs involved in the identified pathways are shown in the lower panel.

**Figure 5.**
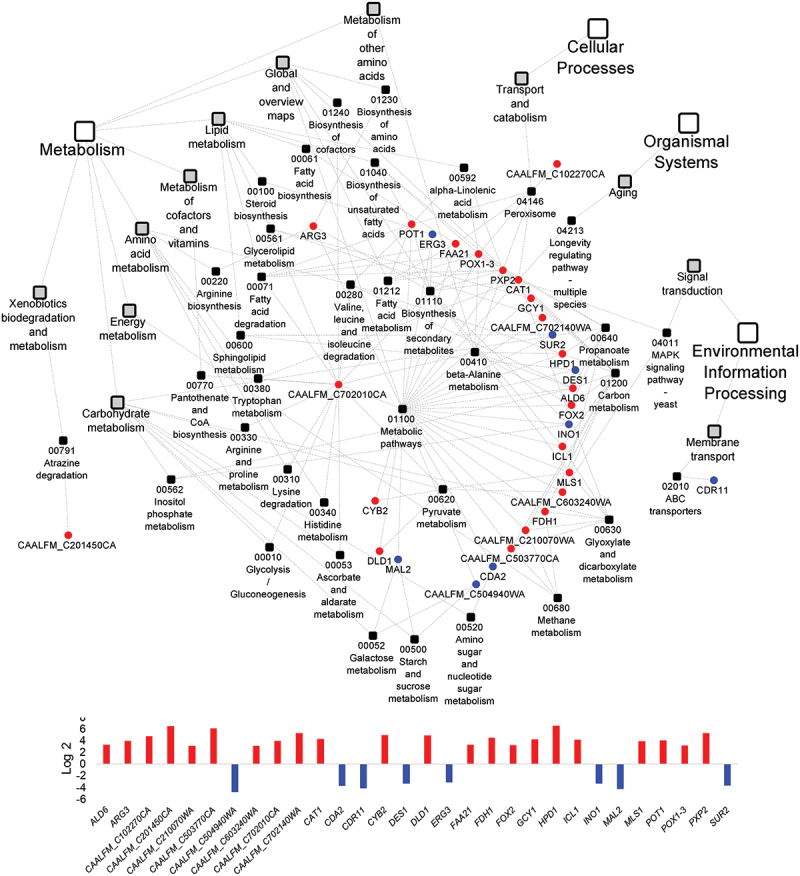
**KEGG pathway network for *C. albicans* differentially expressed genes between the duo-species and single-species biofilms** The genes of *C. albicans* that are differentially expressed between the comparison groups with FDR p-values < 0.05 and log2 fold changes > 3 were defined as *C. albicans* DEGs and listed in Supplementary Table 4. 36 impacted pathways were found for 92 *C. albicans* DEGs. The fold change of the DEGs involved in the identified pathways are shown in the lower panel.

We further performed KEGG pathway analyses to understand the functional association with gene expression. KEGG PATHWAY is a collection of pathway maps representing the knowledge of the molecular interaction, reaction and relation networks for metabolism, genetic information processing, environmental information processing, cellular processes, organismal systems, human diseases and drug development. *mutans* DEGs [> (-) 1 Log2 fold change] and 92 *C. albicans* DEGs [> (-) 3 Log2 fold change] were input into KEGG mapper for analysis. 17 pathways for *S. mutans* and 36 pathways for *C. albicans* were mapped in Figure 4 and Figure 5. KEGG pathway analyses showed that nearly all the *S. mutans* and *C. albicans* DEGs that related to metabolism pathways were up-regulated.

Overall, *S. mutans* genes involved in metabolism were up-regulated, including carbohydrate metabolism, galactose metabolism, glycolysis/gluconeogenesis, and global and overall metabolism (Figure 4). For example, *atpD*, associated with acid stress tolerance response, was 1.7-fold up-regulated. The majority of *C. albicans* DEGs were up-regulated in the following pathways (Figure 5): metabolism, cellular processes, and organismal systems (aging process that was included in the organismal systems was involved). For instance, a 4-fold up-regulation of *ARG3* in the pathway of arginine biosynthesis and a 5.4-fold up-regulation of *PXP2* of fatty acid degradation was seen in duo-species biofilms. A limited number of *C. albicans* DEGs that are associated with environmental process were down-regulated; for instance, *CDR11* was 4.1-fold down-regulated, which is an ATP-binding cassette transporter and drug efflux gene, essential for phenotypic expression. *MAL2*, a *C. albicans* promoter, was 4.2-fold down-regulated in the pathways of galactose metabolism and starch and sucrose metabolism. *MAL2* is inducible by maltose and repressible by glucose [29].

### Dynamic expression of genes of interest in duo-species biofilms and culture media

**Figure 6.**
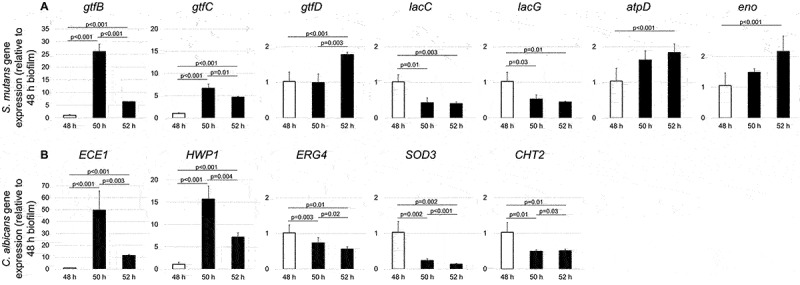
**Dynamic expression of *S. mutans* and *C. albicans* genes in duo-species biofilms** To determine the dynamic transcriptional changes in genes of interest during specific stages of biofilms formation, qRT-PCR was performed for biofilms at 50-h and 52-h. We compared *S. mutans* and *C. albicans* gene expression in the duo-species biofilm (*S. mutans* + *C. albicans*). Panel A indicated dynamic expression of *S. mutans* genes, and panel B indicated dynamic expression of *C. albicans* genes.

Compared to the 48-h biofilms (Figure 6), S. *mutans* genes related to EPS formation (*gtfB, gtfC*, and *gtfD*) were up-regulated after culture medium change; for example, *gtfB* was 26-fold up-regulated at 50-h and 6.5-fold up-regulated at 52-h, when comparing to the value of 48-h. *S. mutans atpD* (related to acid stress tolerance response) and *eno* (related to the degradation of carbohydrates via glycolysis) were also up-regulated at 50-h and 52-h. In contrast, genes related to lactose metabolism (*lacC* and *lacG*) were slightly down-regulated at 50-h and 52-h. Meanwhile, *C. albicans* genes related to *C. albicans* hyphal formation (*ECE1* and *HWP1*) were significantly up-regulated following culture medium changes: *ECE1* with a 50-fold change at 50-h and an 11.5-fold change at 52-h, and *HWP1* with a 15.8-fold change at 50-h and a 7.2-fold change at 52-h. On the contrary, *C. albicans ERG4* (related to antifungal medication resistance), *SOD3* (related to cytotoxic oxygen radicals destroying), and *CHT2* (related to fungal cell wall chitin remodeling) were slightly down-regulated at 50-h and 52-h.

**Figure 7.**
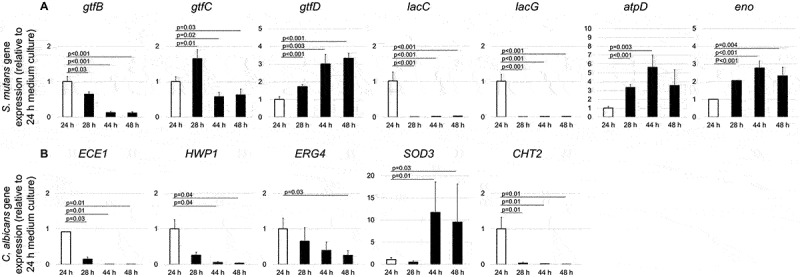
**Dynamic expression of *S. mutans* and *C. albicans* genes in culture medium** To determine the dynamic transcriptional changes in genes of interest during the earlier stages of biofilms formation, qRT-PCR was performed for culture medium at 24-h, 28-h, 44-h, and 48-h. We compared *S. mutans* and *C. albicans* gene expression of the culture medium of duo-species biofilm (*S. mutans* + *C. albicans*). Panel A indicated dynamic expression of *S. mutans* genes, and panel B indicated dynamic expression of *C. albicans* genes.

In addition to assessing gene expression in *S. mutans* and *C. albicans* in biofilm conditions, we further assessed the dynamic changes of these microorganism genes in the culture medium at the early stage of biofilm formation (scheme shown in Figure 1). When the culture medium at the later time points (28-h, 44-h, and 48-h) was compared to the culture medium at 24-h, *S. mutans gtfB, lacC*, and *lacG* were down-regulated, however, *gtfD, atpD*, and *eno* were up-regulated (Figure 7). *gtfC* was up-regulated a short time (28-h) after the culture medium change, followed by a down-regulation at 44-h and 48-h. *S. mutans atpD* (related to acid stress tolerance response) was 5.6-fold up-regulated in the 44-h culture medium. On the contrary, *lacC* and *lacG* (related to galactose metabolism) were significantly down-regulated at all time points after 24-h. For *C. albicans*, the expression of *ECE1, HWP1, ERG4*, and *CHT2* in the culture medium was down-regulated at 28-h, 44-h, and 48-h (Figure 7). Interestingly, *C. albicans SOD3* (related to cytotoxic oxygen radicals destroy) was significantly up-regulated with an 11.8-fold change at 44-h and a 9.5-fold change at 48-h.

## Discussion

### The impact of the duo-species cross-kingdom interplay on *S.* *mutans*

Understanding the molecular basis for microbial interactions is critical to developing a new therapeutic strategy for oral polymicrobial infectious diseases, such as dental caries related to *S. mutans* and *C. albicans* colonization. In recent years, various studies focused on the role/contributions of *S. mutans*-derived exoenzyme glucosyltransferase B (*gtfB*), synergistic carbohydrate metabolism, and enhanced sugar metabolism in *S. mutans* and *C. albicans* mixed-species biofilms. This mixed-species biofilm contains an extensive matrix of extracellular α-glucans, which is produced by *S. mutans gtfB. gtfB* gene readily binds to *C. albicans* cells strongly and stably in an active form [30]. Mannans locates on the outer surface of *C. albicans* cell-wall mediates *gtfB* gene binding, enhancing glucan-matrix production and modulating bacterial-fungal association within the biofilm. *S. mutans*-secreted *gtfB* gene binds to the mannan layer of *C. albicans* to promote extracellular matrix formation and their co-existence within biofilms [31].

In the present study, the dynamic expression of the *gtfB* supports the key role of *gtfB* in developing duo-species biofilms over time. The *gtfB* in biofilms was significantly up-regulated at 50-h and 52-h (compared to 48-h). The highest biofilm *gtfB* expression occurred at 50-h, a short time after the culture medium was changed. Thus, *gtfB* expression might be induced by fresh culture medium, which is nutrient-rich and contains the sucrose necessary for production of α-glucan by *gtfB*. However, *gtfB* was down-regulated in the culture medium at 28-h, 44-h, and 48-h (compared to 24-h), suggesting *gtfB* is more highly expressed in biofilms than in the culture medium at these later time points. In agreement with our observations, *gtfB* expression is typically enhanced in biofilm versus planktonic modes of growth, consistent with its role in biofilm matrix formation [31].

Previous studies demonstrated that *C. albicans* stimulates *S. mutans* micro-colony development via mixed-species biofilm-derived metabolites. Consistently, in our study, all *S. mutans* DEGs (> (-) 1 Log2 fold change) related to metabolism pathways were up-regulated during the formation of duo-species biofilms. For instance, we observed the upregulation of *S. mutans lacC* and *lacG* (galactose metabolism) and *atpD* (related to acid stress tolerance response).

Compared to a previous study that showed the presence of *C. albicans* dramatically altered gene expression in mixed-species biofilms, our results for *lacC* and *lacG* expression was consistent [19]. He et al. revealed elevated pyruvate and galactose metabolism when *S. mutans* and *C. albicans* were grown together, suggesting co-cultivation influenced the carbohydrate utilization by *S. mutans* [19]. Among *S. mutans* DEGs, we found that the amount of up-regulated *S. mutans* DEGs in the duo-species biofilms was 3.8-fold higher than that of the down-regulated DEGs. This observation may indicate enhancement in the metabolism of *S. mutans* when grown with *C. albicans*.

We also found that *S. mutans lytR* and *glnB*, which are involved in a two-component system pathway related to environmental information processing, were up-regulated in duo-species biofilm. The LytR protein, encoded by ORF0317, belongs to the LytR/CpsA/Psr protein family. This family plays a putative role in maintaining cell wall structure. The *lytR* mutant *S. mutans* strain has a defect in cell division, and it shows increased autolysin activity [32]. The transcriptomic up-regulation of *lytR* expression during duo-species biofilms formation in this study indicated a lower autolytic activity and cell-wall re-modeling when co-culturing with *C. albicans*. Therefore, *S. mutans* likely has a more stable cell-wall structure in duo-species biofilms. GlnB belongs to the PII type signal transduction protein family, which regulates enzyme activity and gene expression and is involved in nitrogen regulation/metabolism, as well as glutamine synthesis activities in bacterial species. The PII protein encoded by *glnB* also regulates the expression of the putative ammonium transporter [33]. Our results confirm that when grown together, *C. albicans* augments expression of *S. mutans* genes involved in biofilm formation, virulence expression, and metabolism, which offers a mutual benefit to duo-species biofilm.

### The impact of the duo-species cross-kingdom interplay on *C.* *albicans*

Most studies have focused on the effect of C. albicans on *S. mutans* virulence and biofilm formation. Therefore, we also focused on the molecular changes in *C. albicans* during duo-species interactions. We found that the transcriptomic profile of *C. albicans* was significantly altered when co-cultured with *S. mutans* in duo-species biofilms. Compared to *C. albicans* single species-biofilms, we identified 92 *C. albicans* DEGs (with over 3-fold Log2 fold change) in the duo-species biofilms. The majority of *C. albicans* DEGs that related to metabolism pathways were up-regulated, such as *ARG3, PXP2, and CAT1. ARG3* is related to arginine biosynthesis. The critical roles of the *C. albicans* arginine biosynthesis pathway in its cross-kingdom interactions with *Actinomyces viscosus* in root caries were identified recently, and the study results indicated that targeting this pathway was a practical way to treat root caries caused by multiple species [34]. *PXP2* relates to fatty acid degradation and the peroxisome pathway. The peroxisome plays an essential role in eukaryotic cellular metabolism, including beta-oxidation of fatty acids and detoxification of hydrogen peroxide [35]. *CAT1* is a catalase-specific inhibitor that can suppress the hyphal growth of wild-type cells, and it is also involved in *C. albicans* peroxisome, MAPK signaling, and longevity regulating pathways.

We also pinpointed several down-regulated *C. albicans* genes in duo-species biofilms. For instance, *CDR11* was down-regulated in the pathways of the environmental process. The expression of genes for major ABC transporters (*CDRs*) has been reported as being positively correlated with increased azole resistance in *C. albicans* isolates and is up-regulated during biofilm growth [36]. However, in this study, the transcriptional level of efflux gene *CDR11* was decreased by 4.1-fold in duo-species biofilm, which might suggest that the presence of *S. mutans* represses the drug efflux activity of *C. albicans*, although further investigation is needed. It is plausible that the added benefit of growing with *S. mutans* may circumnavigate the need for high efflux pump expression. Namely, it has been demonstrated that co-culture with *S. mutans* can greatly increase biofilm size and matrix deposition, which acts a diffusion barrier to antimicrobials [37], which may reduce the need to efflux pumps.

In addition, the expression of *C. albicans* hyphal formation *ECE1* and *HWP1* in the culture medium was significantly repressed at 28-h, 44-h, and 48-h (compared to 24-h culture medium), while these genes were significantly induced in biofilms at 50-h and 52-h (compared to 48-h biofilm). This finding indicates that a biofilm condition is favorable for the expression of *ECE1* and *HWP1*. This is consistent with previous findings showing a large portion of the *S.mutans-C. albicans* duo-species biofilm is comprised of *C. albicans* hyphae [12].

*SOD3*, a *C. albicans* gene associated with cytotoxic oxygen radical defense, was significantly induced in the culture medium at 44-h and 48-h, at a relative long period after culture medium change. This may reflect a response to increasing levels of oxidative stress in the culture media as these two organisms are growing together. Microbial respiration is a significant source of oxidative radicals [38]. *SOD3* overexpression from a conditional promoter could substantially restore the cells’ oxidative stress resistance [39]. Co-culturing with *S. mutans* at a relatively long-time stage might enhance *C. albicans*’ tolerance for oxidative stress.

Overall, our results show the effects of duo-species biofilm represent a ‘two-way street’ where the presence of *S. mutans* influences *C. albicans* metabolism and behavior and vice versa. The following limitations are recognized with the intriguing finding: although our study results indicated the interactions between *S. mutans* and *C. albicans* during the formation of the duo-species biofilms at the transcriptomic level, further confirmation of these interactions needs to be conducted with mutant strains with the deletion of specific genes. Second, our study has not assessed the interactions at the protein level expression, which needs to be confirmed in the future. Third, cariogenic assessment of the duo-species interactions in the animal models was not conducted in our study; however, it deserves further investigation. This study is among the first to characterize the molecular mechanisms of duo-species biofilm formation and represents a launching point for future investigation.

## Conclusions

The cross-kingdom interplay characterized here impacts *S. mutans* and impacts *C. albicans* metabolism, cellular processes, and organismal systems pathways. Furthermore, expression is not static and changes over time with the relative level of available carbohydrate (e.g. sucrose) and development stage. There are distinct differences between adherent biofilm cells and the planktonic milieu. It is clear from this study and others that the relationship between these two organisms is unique and enhances overall biofilm formation and virulence of these organisms, which likely contributes to ECC. Further targeting these interactions could be an ideal strategy for combating ECC.

## Supplementary Material

Supplemental Material
