# Supplementary material for: Dual transcriptome of Streptococcus mutans and Candida albicans interplay in biofilms
Source: J Oral Microbiol. 2022 Nov 9;15(1):2144047. doi: 10.1080/20002297.2022.2144047 (PMC9662060; doi:10.1080/20002297.2022.2144047)
Supplement: Supplemental Material [file ZJOM_A_2144047_SM8813.docx]

**Title: Dual transcriptome of *Streptococcus mutans* and *Candida albicans* interplay in biofilms**

**Supplemental materials**


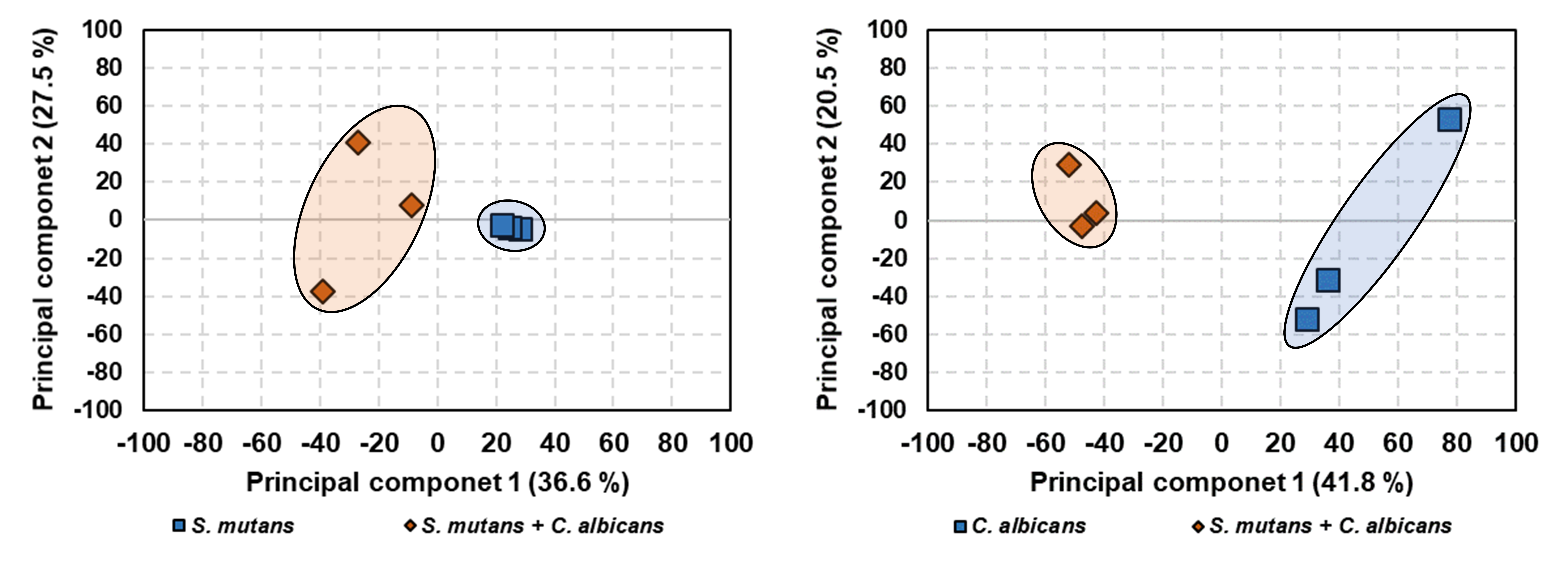


**Figure S1. Principal Component Analysis (PCA) for RNA samples from 48h biofilm**


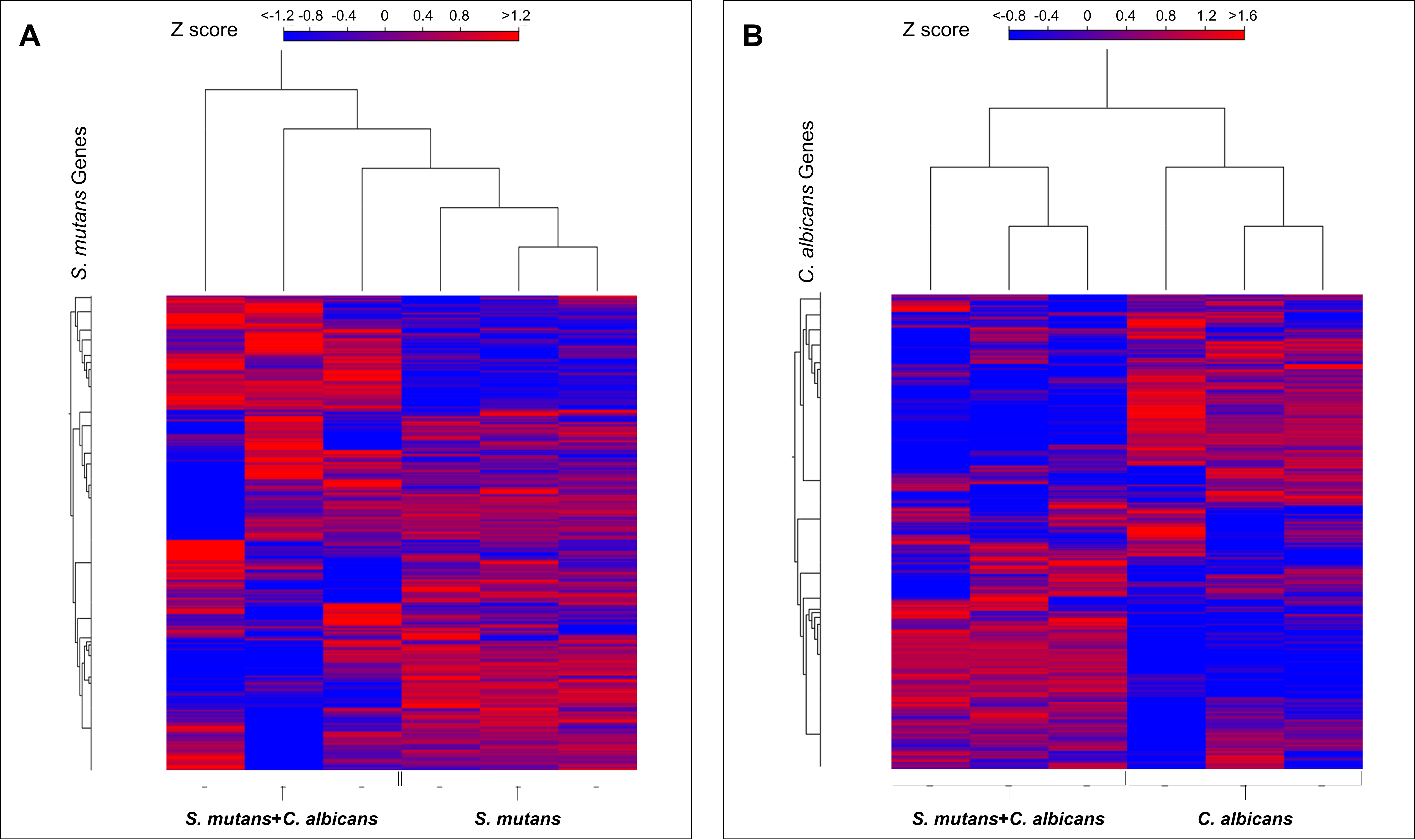


**Figure S2. Hierarchical clustering analysis for RNAseq samples from 48h biofilm**

Based on gene expression in RNA-seq, hierarchical clustering of features presented the similarity in expression profiles of the features over groups. (A) hierarchical clustering of *S. mutans* genes in the duo-species biofilm and *S. mutans* single species biofilm. (B) hierarchical clustering of *C. albicans* genes in the duo-species biofilm and *C. albicans* single species biofilm.


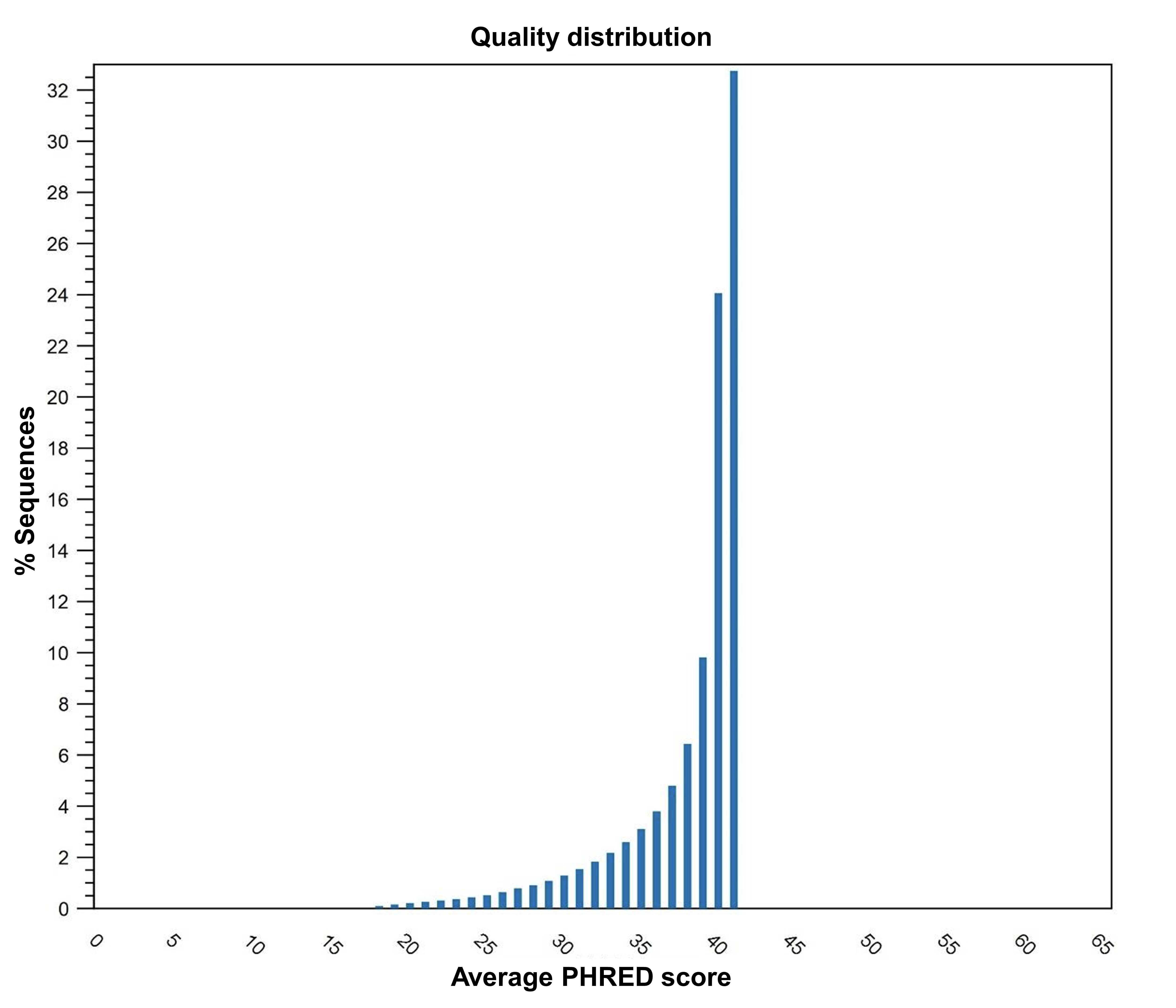


**Figure S3. Q-Score distribution of RNA samples from 48h biofilm**


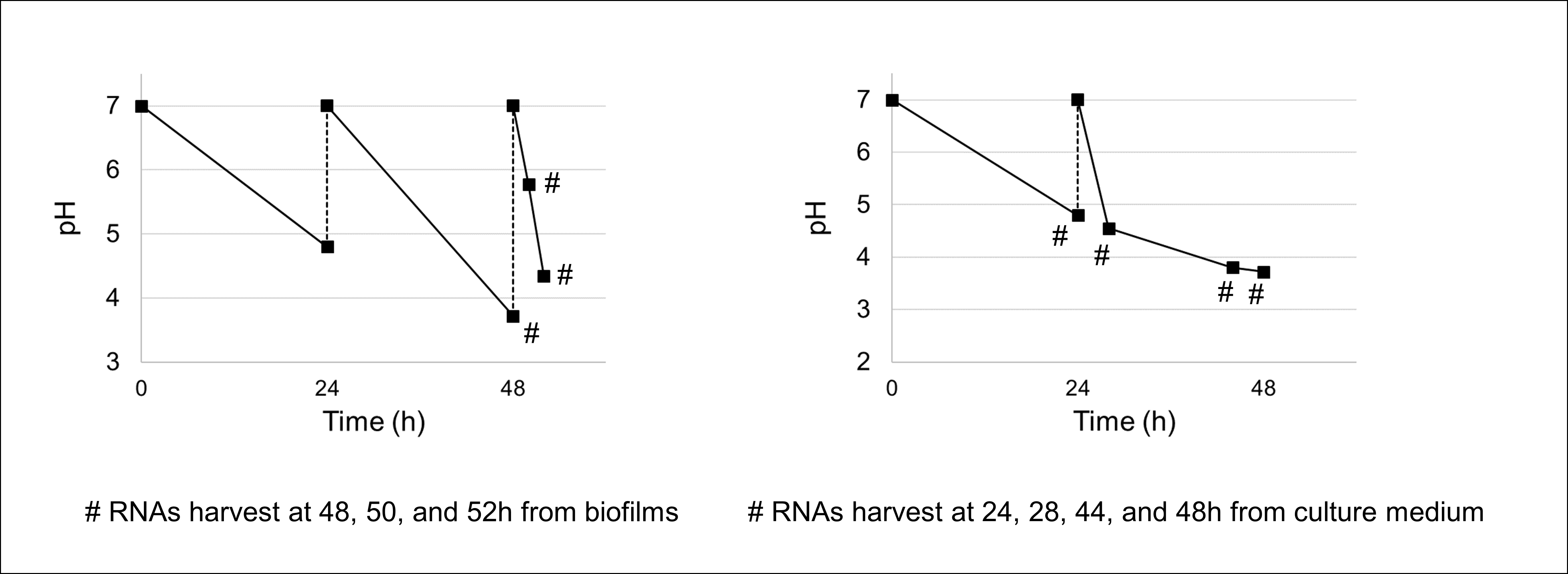


**Figure S4. pH changes in the culture medium of duo-species biofilms**

| **Table S1. Primers used in RT-qPCR** | | | | |
| --- | --- | --- | --- | --- |
| Genes | Primers | Sequence | Amplicon size (bp) | Source or References |
| *gyrA* | Sm_gyrA_F | CCAAGAATCTGCTGTCCG | 111 | [1] |
|  | Sm_gyrA_R | TTGCGACTATCTGCTATGTG |  |  |
| *gtfB* | Sm_gtfB_F | AGCAATGCAGCCATCTACAAAT | 96 | [2] |
|  | Sm_gtfB_R | ACGAACTTTGCCGTTATTGTCA |  |  |
| *gtfC* | Sm_gtfC_F | CTCAACCAACCGCCACTGTT | 91 | [2] |
|  | Sm_gtfC_R | GGTTTAACGTCAAAATTAGCTGTATTAGC |  |  |
| *gtfD* | Sm_gtfD_F | CACAGGCAAAAGCTGAATTAACA | 81 | [2] |
|  | Sm_gtfD_R | GAATGGCCGCTAAGTCAACAG |  |  |
| *atpD* | Sm_atpD_F | TGTTGATGGTCTGGGTGAAA | 176 | [3] |
|  | Sm_atpD_R | TTTGACGGTCTCCGATAACC |  |  |
| *eno* | Sm_eno_F | CAGCGTCTTCAGTTCCATCA | 194 | [3] |
|  | Sm_eno_R | TCACTCAGATGCTCCAATCG |  |  |
| *lacG* | Sm_lacG_F | ATTGGATGCGTGCTTTTGATGG | 94 | [4] |
|  | Sm_lacG_R | CGACCGACACCCTTAATCTGG |  |  |
| *lacC* | Sm_lacC_F | GCTGGAATTACATCGGCTCTTGC | 157 | [4] |
|  | Sm_lacC_R | CCTCCGCTACCTCAATTTGTTGG |  |  |
|  |  |  |  |  |
| *ACT1* | Ca_ACT1_F | TGCTCCAGAAGAACACCCA | 182 | [5] |
|  | Ca_ACT1_R | CACCTGAATCCAAAACAATACCAGT |  |  |
| *HWP1* | Ca_HWP1_F | TGGTGCTATTACTATTCCGG | 182 | [6] |
|  | Ca_HWP1_R | CAATAATAGCAGCACCGAAG |  |  |
| *ECE1* | Ca_ECE1_F | GCTGGTATCATTGCTGATAT | 168 | [6] |
|  | Ca_ECE1_R | TTCGATGGATTGTTGAACAC |  |  |
| *CHT2* | Ca_CHT2_F | TTGGGATGCTTCTGGGGCTT | 111 | This study |
|  | Ca_CHT2_R | GCAGAAGAAGATGGGGCAACAC |  |  |
| *ERG4* | Ca_ERG4_F | TCAAATGTGCCAATGGTTCT | 101 | [7] |
|  | Ca_ERG4_R | AGCCCAAGTCAATGTTTGAA |  |  |
| *SOD3* | Ca_SOD3_F | CAGTATGGGTCTGTTTCAAACCTTA | 211 | [8] |
|  | Ca_SOD3_R | GATATTGCAAGTAGTACGCATGTTC |  |  |

| References: | |
| --- | --- |
| [1] | He J, Kim D, Zhou X, Ahn SJ, Burne RA, Richards VP et al (2017). RNA-Seq Reveals Enhanced Sugar Metabolism in *Streptococcus mutans* Co-cultured with *Candida albicans* within Mixed-Species Biofilms. Front Microbiol 8: 1036. |
| [2] | Ahn SJ, Lemos JAC, Burne RA (2005). Role of *HtrA* in growth and competence of *Streptococcus mutans* UA159. Journal of Bacteriology 187: 3028-3038. |
| [3] | Xu X, Zhou XD, Wu CD (2011). The Tea Catechin Epigallocatechin Gallate Suppresses Cariogenic Virulence Factors of *Streptococcus mutans*. Antimicrobial Agents and Chemotherapy 55: 1229-1236. |
| [4] | Zeng L, Das S, Burne RA (2010). Utilization of Lactose and Galactose by *Streptococcus mutans*: Transport, Toxicity, and Carbon Catabolite Repression. Journal of Bacteriology 192: 2434-2444. |
| [5] | Branco J, Martins-Cruz C, Rodrigues L, Silva RM, Araujo-Gomes N, Goncalves T et al (2021). The transcription factor Ndt80 is a repressor of *Candida* parapsilosis virulence attributes. Virulence 12: 601-614. |
| [6] | Wang S, Wang QY, Yang EC, Yan L, Li T, Zhuang H (2017). Antimicrobial Compounds Produced by Vaginal *Lactobacillus crispatus* Are Able to Strongly Inhibit *Candida albicans* Growth, Hyphal Formation and Regulate Virulence-related Gene Expressions. Front Microbiol 8: 11. |
| [7] | Dorsaz S, Snaka T, Favre-Godal Q, Maudens P, Boulens N, Furrer P et al (2017). Identification and Mode of Action of a Plant Natural Product Targeting Human Fungal Pathogens. Antimicrobial Agents and Chemotherapy 61. |
| [8] | Li CX, Gleason JE, Zhang SX, Bruno VM, Cormack BP, Culotta VC (2015). *Candida albicans* adapts to host copper during infection by swapping metal cofactors for superoxide dismutase. Proceedings of the National Academy of Sciences of the United States of America 112: E5336-E5342. |

**Table S2. Differential gene expression of *S. mutans* grown in duo-species biofilm vs. single-species biofilm.**

**Significant genes ( >(-)1 Log2 fold change and FDR p value<0.05) that fit KEGG pathways are shown below.**

| Duo-species biofilm: *S. mutans*+*C. albicans*  Single-species biofilm: *S. mutants* | | | | | | | | | |
| --- | --- | --- | --- | --- | --- | --- | --- | --- | --- |
| **GeneID** | **Gene name** | **Log2 fold change** | **FDR p value** | **Description** | | | | | |
| SMU_1533 | *atpB* | 1.049145471 | 0.02415125 | F0F1 ATP synthase subunit A | |  |  |  |  |
| SMU_1528 | *atpD* | 1.692470475 | 8.30964E-05 | aminoacyl-tRNA hydrolase | |  |  |  |  |
| SMU_1123 | *deoC* | 1.025421101 | 1.48468E-08 | deoxyribose-phosphate aldolase | | |  |  |  |
| SMU_1657c | *glnB* | 1.029699249 | 0.048973218 | P-II family nitrogen regulator | |  |  |  |  |
| SMU_1271 | *hisG* | 1.122969717 | 0.028204463 | ATP phosphoribosyltransferase | | |  |  |  |
| SMU_1496 | *lacA* | 1.728634558 | 0.020187957 | galactose-6-phosphate isomerase subunit LacA | | | |  |  |
| SMU_1494 | *lacC* | 1.990234027 | 3.55576E-05 | tagatose-6-phosphate kinase | |  |  |  |  |
| SMU_1493 | *lacD* | 1.547840706 | 0.001028378 | tagatose-bisphosphate aldolase | | |  |  |  |
| SMU_1668 | *livH* | -1.77240564 | 2.17136E-07 | branched-chain amino acid ABC transporter permease | | | | |  |
| SMU_576 | *lytR* | 1.006429025 | 0.01247218 | response regulator |  |  |  |  |  |
| SMU_1570 | *malG* | 1.028006366 | 0.035825147 | sugar ABC transporter permease | | |  |  |  |
| SMU_1421 | *pdhC* | 1.788251952 | 0.00051709 | 2-oxo acid dehydrogenase subunit E2 | | |  |  |  |
| SMU_871 | *pfkB* | 1.798005008 | 0.033981049 | 1-phosphofructokinase | |  |  |  |  |
| SMU_1234 | *rpiA* | 1.094215448 | 0.000692046 | ribose-5-phosphate isomerase RpiA | | |  |  |  |

**Table S3. Differential gene expression of *C.albicans* grown in duo-species biofilm vs. single-species biofilm.**

**Significant genes ( >(-)3 Log2 fold change and FDR p value<0.05) that fit KEGG pathways are shown below.**

| Duo-species biofilm: *S. mutans*+*C. albicans*  Single-species biofilm: *C. albicans* | | | | | | | | | | |  |  |  |
| --- | --- | --- | --- | --- | --- | --- | --- | --- | --- | --- | --- | --- | --- |
| **GeneID** | **Gene name** | **Log2 fold change** | **FDR p value** | **Description** | | | | | | |  |  |  |
| CAALFM_C405130CA | *ALD6* | 3.333077428 | 0 | Ald6p |  |  |  |  |  |  |  |  |  |
| CAALFM_C603230WA | *ARG3* | 3.976371902 | 0 | ornithine carbamoyltransferase | | | |  |  |  |  |  |  |
| CAALFM_C102270CA | *CAALFM_C102270CA* | 4.764111776 | 0 | hypothetical protein | | |  |  |  |  |  |  |  |
| CAALFM_C201450CA | *CAALFM_C201450CA* | 6.54123032 | 7.62941E-07 | cyanamide hydratase | | |  |  |  |  |  |  |  |
| CAALFM_C210070WA | *CAALFM_C210070WA* | 3.135768278 | 0.00022778 | hypothetical protein | | |  |  |  |  |  |  |  |
| CAALFM_C503770CA | *CAALFM_C503770CA* | 6.150124269 | 0.014157517 | hypothetical protein | | |  |  |  |  |  |  |  |
| CAALFM_C504940WA | *CAALFM_C504940WA* | -4.72611789 | 0 | hypothetical protein | | |  |  |  |  |  |  |  |
| CAALFM_C603240WA | *CAALFM_C603240WA* | 3.131742942 | 0.001933436 | methylglyoxal reductase (NADPH-dependent) | | | | |  |  |  |  |  |
| CAALFM_C702010CA | *CAALFM_C702010CA* | 4.024117117 | 0.000344728 | hypothetical protein | | |  |  |  |  |  |  |  |
| CAALFM_C702140WA | *CAALFM_C702140WA* | 5.330695633 | 0.045221455 | hypothetical protein | | |  |  |  |  |  |  |  |
| CAALFM_C106810WA | *CAT1* | 4.331333975 | 0 | catalase A | |  |  |  |  |  |  |  |  |
| CAALFM_CR00300WA | *CDA2* | -3.661023667 | 0.037270727 | chitin deacetylase | |  |  |  |  |  |  |  |  |
| CAALFM_C304070CA | *CDR11* | -4.061052537 | 0 | Cdr11p |  |  |  |  |  |  |  |  |  |
| CAALFM_C113630WA | *CYB2* | 5.015343738 | 0 | Cyb2p |  |  |  |  |  |  |  |  |  |
| CAALFM_C505480WA | *DES1* | -3.308737328 | 0 | Des1p |  |  |  |  |  |  |  |  |  |
| CAALFM_C202980CA | *DLD1* | 4.918754834 | 0 | Dld1p |  |  |  |  |  |  |  |  |  |
| CAALFM_C104770CA | *ERG3* | -3.121698141 | 0 | C-5 sterol desaturase | | |  |  |  |  |  |  |  |
| CAALFM_C302810CA | *FAA21* | 3.353752287 | 0 | medium-chain fatty acid-CoA ligase | | | |  |  |  |  |  |  |
| CAALFM_CR05170CA | *FDH1* | 4.510385612 | 0 | formate dehydrogenase (NAD+) | | | |  |  |  |  |  |  |
| CAALFM_C300810CA | *FOX2* | 3.257800764 | 0 | bifunctional hydroxyacyl-CoA dehydrogenase/enoyl-CoA hydratase | | | | | | | | | |
| CAALFM_C307340WA | *GCY1* | 4.247086031 | 0 | glycerol 2-dehydrogenase (NADP(+)) | | | |  |  |  |  |  |  |
| CAALFM_C602890CA | *HPD1* | 6.56081398 | 5.31E-15 | Hpd1p |  |  |  |  |  |  |  |  |  |
| CAALFM_C104500WA | *ICL1* | 4.170609291 | 0 | isocitrate lyase 1 | |  |  |  |  |  |  |  |  |
| CAALFM_CR10100CA | *INO1* | -3.324122252 | 0 | inositol-3-phosphate synthase | | | |  |  |  |  |  |  |
| CAALFM_CR10790WA | *MAL2* | -4.202809035 | 0 | oligo-1,6-glucosidase IMA1 | | |  |  |  |  |  |  |  |
| CAALFM_C109690WA | *MLS1* | 3.938773949 | 0 | malate synthase | |  |  |  |  |  |  |  |  |
| CAALFM_CR00150CA | *POT1* | 4.077483275 | 0 | acetyl-CoA C-acyltransferase | | |  |  |  |  |  |  |  |
| CAALFM_C301960CA | *POX1-3* | 3.183896196 | 0 | acyl-CoA oxidase | |  |  |  |  |  |  |  |  |
| CAALFM_C301930WA | *PXP2* | 5.354443242 | 0 | Pxp2p |  |  |  |  |  |  |  |  |  |
| CAALFM_C202860WA | *SUR2* | -3.612366836 | 0 | sphingosine hydroxylase | | |  |  |  |  |  |  |  |
|  |  |  |  |  |  |  |  |  |  |  |  |  |  |
| All FDR p value that less than 1E-15 shown as 0. | | |  |  |  |  |  |  |  |  |  |  |  |
